# Home Parenteral Nutrition in Patients with Advanced Cancer: Quality Outcomes from a Centralized Model of Care Delivery

**DOI:** 10.3390/nu14163379

**Published:** 2022-08-17

**Authors:** Maja Kopczynska, Antje Teubner, Arun Abraham, Michael Taylor, Ashley Bond, Andrew Clamp, Rebecca Wight, Zena Salih, Jurjees Hasan, Claire Mitchell, Gordon C. Jayson, Simon Lal

**Affiliations:** 1 Intestinal Failure Unit, Salford Royal NHS Foundation Trust, Salford M6 8HD, UK; 2 School of Health Sciences, University of Manchester, Manchester M13 9PL, UK; 3 Department of Medical Oncology, The Christie NHS Foundation Trust, Manchester M20 4BX, UK

**Keywords:** advanced cancer, home parental nutrition, outcomes

## Abstract

Lack of expertise in home parenteral nutrition (HPN) management has been reported as a barrier to its initiation in patients with advanced cancer (AC), and there are limited data describing hospital readmissions and HPN-related complications. We aimed to assess a centralized approach for managing HPN in AC and evaluate associated outcomes, including hospital readmissions and HPN-related complications. This was a cohort study of adults with AC requiring palliative HPN between 2010–2018 at a tertiary intestinal failure (IF) center, primarily utilizing a centralized model of HPN oversight to discharge patients remotely from an oncology center to their homes over a wide geographic area. A total of 126 patients were included, with a median distance between the patient’s home and the IF center of 17.5 km (IQR 10.9–39.1; maximum 317.4 km). A total of 28 (22%) patients experienced at least one HPN-related complication, the most common being a central venous catheter (CVC) occlusion and electrolyte abnormalities. The catheter-related bloodstream infection (CRBSI) rate was 0.49/1000 catheter days. The CVC type, administration of concomitant chemotherapy via a distinct CVC lumen separate from PN, venting gastrostomy and distance between the patient’s home and the IF center were not associated with CRBSI or mechanical CVC complications. A total of 82 (65.1%) patients were readmitted while on HPN, but only 7 (8.5%) of these readmissions were HPN-related. A total of 44 (34.9%) patients died at home, 41 (32.5%) at a hospice and 41 (32.5%) in a hospital. In conclusion, this study demonstrates that a centralized approach to IF care can provide HPN to patients over a large geographical area while maintaining low HPN-related complications that are comparable to patients requiring HPN for benign conditions and low hospital readmission rates.

## 1. Introduction

Cancer is among the leading causes of morbidity and mortality worldwide, and the number of new cases is expected to rise significantly [1]. In parallel, patients with advanced disease stages may survive for increasing lengths of time, such that associated malnutrition often becomes a significant determinant of their outcome. Indeed, malnutrition is observed in up to 80% of patients with advanced cancer (AC) [2]. The reason for its development is multifactorial, with decreased dietary intake, cancer cachexia, side-effects of chemotherapy, cancer-related intestinal obstruction and complications from surgical intervention all likely contributing [3]. Malnutrition impacts on survival, quality of life, performance status and the ability to tolerate systemic anti-cancer therapy [3,4]. It may, therefore, be reasonable to assume that the administration of nutrients to such malnourished patients should improve long-term energy homeostasis and patient outcomes. However, the role of home parenteral nutrition (HPN) in patients with AC remains controversial [5,6,7].

HPN involves the administration of intravenous solutions containing electrolytes, energy, protein, vitamins and micronutrients, which are usually given intermittently over a 10–14 h overnight period via a central venous catheter (CVC). HPN use in patients with AC differs from one country to another, accounting for around 60% of all HPN indications in the Netherlands and Italy but less than 25% in the UK [8], with intermediate rates in other European countries and the USA [9,10]. The differences in international attitudes and experiences towards the use of HPN in patients with AC have been highlighted in a recent international survey of multi-disciplinary clinicians [11], with the decision to start HPN in patients with AC requiring consideration not only of the clinical parameters but also ethical questions, with the exploration of patients’ preferences being fundamental [1,11]. Notably, logistical issues were highlighted in the international survey as significant barriers to the commencement of HPN, including a lack of local expertise, as well as a lack of funding or community services for HPN administration [11]. Indeed, the organization of HPN delivery can vary between and within countries, with HPN being initiated by oncologists in some hospitals or via larger, centralized intestinal failure (IF) multi-disciplinary teams in others.

Quality outcomes associated with IF and HPN care delivery include CVC-related complication and hospital readmission rates [12,13]. Notably, it has been observed that centralized care and provision of HPN by tertiary centers is associated with reduced HPN-related complication rates [12]. Establishing collaborations between IF centers and oncology departments could facilitate better patient care in addition to enabling remote discharge and monitoring of patients, while also helping to overcome any logistical barriers to HPN initiation that may relate to a lack of clinician experience or service infrastructure [14]. Nevertheless, the recent literature describes uncertainty about whether HPN leads to improvement in the survival or quality of life of patients with AC [6,7]. Additionally, there are limited data on the burden of HPN-related complications or the impact of concomitant chemotherapy on their development, as well as on the investigation of readmission rates or places of death in this patient cohort, all of which can be key determinants of the quality of life in this cohort of patients.

The aim of this study was, therefore, to assess the service delivery of HPN for patients with AC via tertiary IF and oncology centers, as well as to investigate patient outcomes, including survival, HPN-related complications and hospital readmissions.

## 2. Materials and Methods

### 2.1. Study Design

This was a cohort study of adult patients with AC initiated on palliative HPN between 2010–2018 at a tertiary IF reference center or at a specialized oncology hospital, primarily via a remote discharge pathway, details of which are described elsewhere [14]. In brief, after receiving a referral from the oncology center, which was located at a separate hospital around 15 km from the IF center, the team at the IF center evaluated the clinical, nutritional and biochemical status of the patients, as well as the appropriateness of the central venous access and generated a PN prescription. Concurrently, an HPN homecare company was appointed to facilitate the set-up of the necessary HPN equipment at the patient’s home and the establishment of nurse-led CVC care or patient/relative training for PN administration at home, as appropriate, depending on the patient’s needs. Once these steps were in place and the patient was optimized for discharge from the oncology team, the patient commenced HPN treatment [14]. Patients with AC who were admitted to the IF center were discharged home directly. All consecutively included patients were identified from a prospectively maintained database. The inclusion criteria were as follows: age > 18 years; patients receiving HPN for a diagnosis of advanced cancer; HPN administered via a central venous catheter. Patients with missing data due to a lack of clinical records (*n* = 8) were excluded. Patients were followed from their first discharge with HPN until death.

### 2.2. Data Collection

Data were collected on patient demographics (including distance from the patient’s home to the IF center), clinical characteristics, HPN-related factors, including start date of HPN, total duration of time on HPN, indication for HPN, CVC care (involving nurse or patient/family HPN connections and disconnections) and nutritional status. HPN complications included the following: catheter-related blood stream infections (CRBSI), CVC thrombosis, mechanical CVC complications (CVC occlusion and CVC dislodgement) [15] and significant electrolyte abnormalities. Data on hospital readmissions as well as the date and place of death were also collected.

This study was approved by the local Research and Innovation Department (Reference No: S21HIP07) as a Service Evaluation. The confidentiality and anonymity of the included patients were maintained, and viewing of the medical records was undertaken by a direct member of the clinical team; therefore, informed consent was not required as per the Data Protection and Caldicott Framework.

### 2.3. Statistical Analysis

The categorical variables are described as proportions and the continuous variables are described as mean ± SD or median (IQR, range). The analyses were performed using a chi-square test for the categorical variables and a Mann–Whitney U or a Kruskal–Wallis test for the non-parametric continuous variables to compare different groups of subjects. A univariate Poisson regression was used to determine which variables were associated with CRBSI and mechanical CVC complications. The unit of exposure (HPN days) was added as an “offset” variable in all of the Poisson regression analyses. The Pearson dispersion statistic was used to evaluate the presence of overdispersion in the models, and in the cases of dispersion greater than 1, scaling of standard errors and robust variance estimators were used to adjust for the overdispersion. The associations with outcomes were expressed as incidence rate ratios (IRRs). The statistical software used was R Version 4.0.3.

## 3. Results

### 3.1. Patient Demographics and Clinical Characteristics

**Figure 1:**
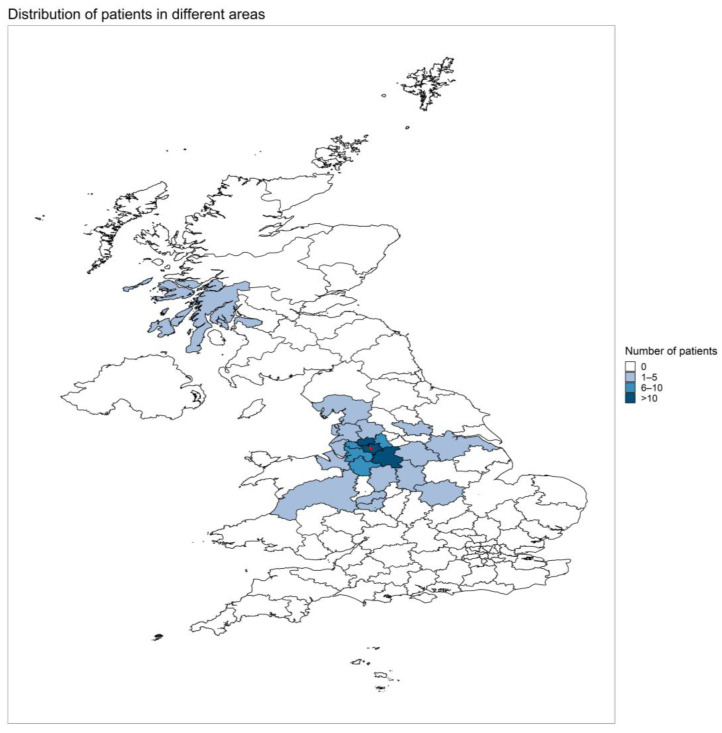
Map of the UK with the distribution of patients with AC receiving HPN under the care of the IF center. The red dot marks the location of the IF center.

In total, 126 patients were included in the analysis. The median patient age at HPN initiation was 58 years (IQR 49–69). The most common indication for HPN initiation was malignant bowel obstruction in 117/126 (93%) patients. Sixty (48%) patients underwent venting gastrostomy insertion prior to or during the HPN treatment. The vast majority of patients (114/126, 97%) required HPN seven nights per week. Patient demographics and clinical characteristics, including underlying malignancy, are summarized in Table 1. Most patients (94/126, 74.6%) were established on HPN via the remote discharge pathway from the oncology center, with the remainder being discharged directly from the IF center. The median distance between the patient’s home and the IF center was 17.5 km (IQR 10.9–39.1), with the greatest distance being 317.4 km. Areas of the HPN provision in relation to the IF center are presented in Figure 1.

### 3.2. Changes over the Study Period

The number of patients with AC referred for HPN increased from 19 in the years 2010–2012 to 24 in 2013–2015 and to 83 in 2016–2018. The median patient age at HPN initiation also increased over the study period (49, 56 and 61 years, respectively). The indications for HPN remained stable over the study period, with bowel obstruction being the most common indication throughout (84.2%, 87.5% and 96.4% of patients, respectively). The median time from referral to discharge from hospital on HPN reduced from 37 days in the years 2010–2012 to 18 days in 2013–2015 and to 16 days in 2016–2018.

### 3.3. Catheter Characteristics

For the majority of patients (114/126, 90%), the HPN administration was undertaken by homecare nurses, compared to self-administration or via a relative or carer (see Table 2). More patients had a tunnelled CVC than a peripherally inserted central catheter (PICC). In total, 35/126 (28%) patients received concomitant systemic anti-cancer therapy (SACT), with 13/126 (10%) patients using the same CVC for the delivery of both treatments, but always via a distinct lumen, separate from the PN.

### 3.4. HPN Complications

In total, 28/126 (22%) patients experienced at least one complication associated with the HPN treatment. The most common complication was CVC occlusion in 13/126 (10%) patients, followed by electrolyte abnormalities in 10/126 (7.9%), CVC dislodgement in 8/126 (6.3%), CRBSI in 4/126 (3.2%) and CVC thrombosis in 1/126 (0.8%) patients. In total, there were eight episodes of CRBSI experienced by four patients. This corresponded to a CRBSI rate of 0.49 episodes per 1000 catheter days. A Poisson regression evaluating the factors associated with CRBSI is shown in Table 3. All CRBSIs occurred in patients with tunnelled CVCs. For patients with CRBSIs, CVC salvage was attempted and it was successful in three out of eight CRBSI episodes, with the remaining episodes requiring a CVC change. Poisson regressions evaluating the factors associated with mechanical CVC complications are presented in Table 4. The catheter types and lumen numbers, the administration of concomitant SACT via the same CVC as HPN (via a distinct lumen, separate from the PN), the presence of a venting gastrostomy and the distance from the patient’s home to the IF center were not associated with an increased likelihood of CRBSI or mechanical CVC complications.

### 3.5. Readmission to Hospital

Overall, 82/126 (65.1%) patients were readmitted during their course of HPN. In total, 47/126 (57.3%) patients required hospital readmission due to cancer-related reasons, 7/126 (8.5%) patients were readmitted due to HPN-related complications, 7/126 (8.5%) due to problems with venting gastrostomy and 21/126 (25.6%) due to other reasons. Patient location did not impact on the likelihood of readmissions, with 74.5% living within 15 km of the IF center requiring readmission, in comparison to 60.0% of those living within 15–30 km and 57.5% over 30 km away (*p* = 0.18).

### 3.6. Survival Analysis

**Figure 2:**
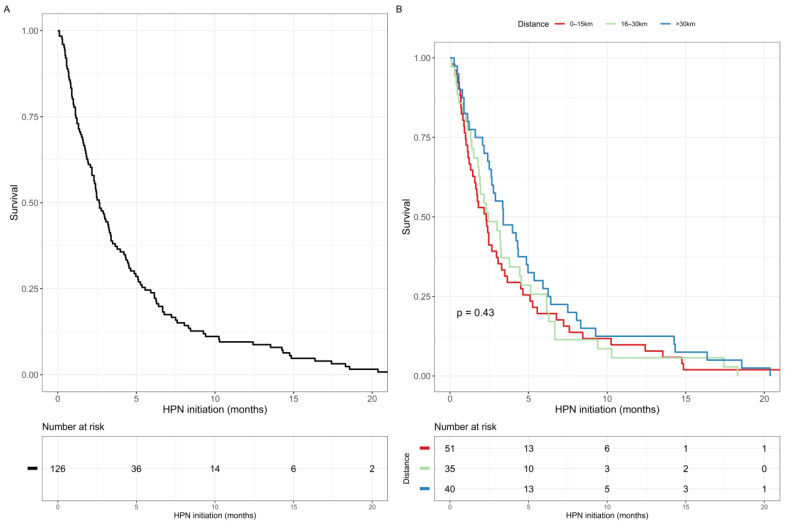
Survival curve for the patient cohort. (**A**) Overall survival, (**B**) Survival stratified by the distance from the patient’s home to the IF center.

**Figure 3:**
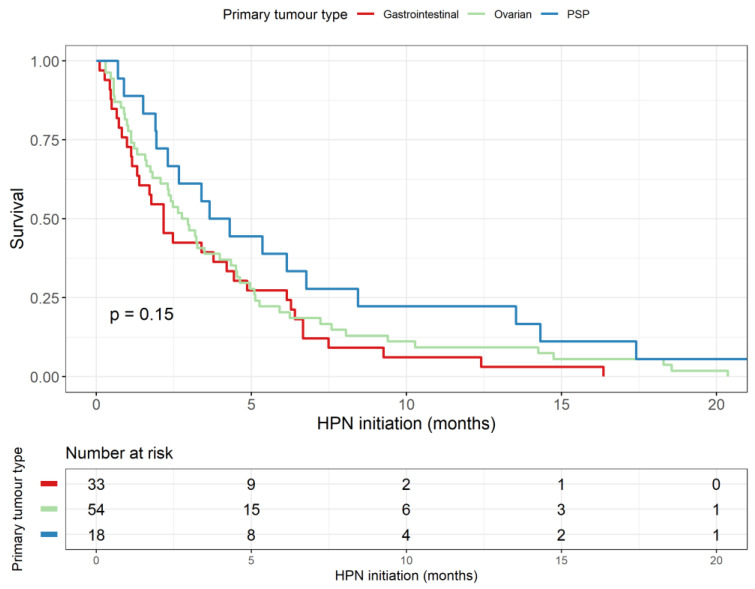
Survival curve stratified by the primary cancer types. PSP, pseudomyxoma peritonei.

The median survival time from HPN initiation was 2.64 months (95% CI 2.17–3.38). The survival curve is presented in Figure 2A. Patient location did not impact on their survival (shown in Figure 2B). The survival curve for the main primary cancer types (ovarian, pseudomyxoma peritonei and gastrointestinal) is presented in Figure 3. For patients with ovarian cancers, the median overall survival was 2.9 months (95% CI 2.1–4.5); for patients with pseudomyxoma peritonei, it was 3.9 months (95% CI 2.3–13.5); for patients with gastrointestinal cancer, the median overall survival was 2.2 months (95% CI 1.3–4.9). In total, 44/126 (34.9%) patients died at home, 41/126 (32.5%) died at a hospice and 41/126 (32.5%) in a hospital.

## 4. Discussion

This is one of the largest single-center series reporting service provision, complications and outcomes, including readmission details, of patients with AC receiving HPN. The novelty of this study is the demonstration that with the centralization of IF care, HPN treatment can be provided over a large geographical area without detriment to patient outcomes. The low HPN-related complication and low HPN-related readmission rates shown in this study, as well as two-thirds of patients dying outside of the hospital, all show that with this centralized approach, good quality outcomes can be achieved in patients with AC on HPN in their last months.

Maintaining a good quality of life is paramount in patients with AC and, therefore, it is important to ensure that the treatment benefits are not overwhelmed by harm. In our cohort, the majority of patients did not experience any complications associated with the HPN treatment and had a low CRBSI rate of 0.49 episodes per 1000 catheter days, which is comparable to patients requiring HPN for benign conditions [16]. Importantly, this is the first study to demonstrate that the use of concomitant systemic anti-cancer therapy did not have a negative impact on the rates of both CRBSI and mechanical CVC complications, even if both treatments were administered via the same CVC. This provides reassurance that with appropriate catheter care and the use of a distinct lumen, separate from the PN, patients can benefit from the HPN treatment with a minimized burden of potential complications associated with it.

Only seven (8.5%) patients required readmission for HPN-related reasons, similar to patients requiring admission due to issues with venting gastrostomy, an intervention recommended by consensus guidelines for those with malignant bowel obstruction [17]. Avoiding HPN-related admissions, together with efficient discharge pathways, is essential for improving patient quality of life and enabling patients to spend more time at home. Notably, in our cohort, avoidance of hospital admissions was maintained for many patients until death, with two-thirds dying either at home or at a hospice.

Discharge on HPN can be clinically and logistically complex [18]. The lack of expertise in establishing HPN for patients with AC was reported as one of the main barriers to effective service provision for these patients in a recent multi-centered European survey [11]. Moreover, a recent study by Mundi et al. showed that a large proportion of patients in the USA were located at a significant distance from their IF center (over 100 miles), indicating a substantial inequality in healthcare coverage throughout the country [19]. Centralization of care has already been shown to improve the outcomes of patients requiring HPN for benign conditions, with an increased number of patients treated in a center being associated with lower mortality and HPN-related complication rates [12]. Our data show that centralized HPN care delivery for patients with malignancy can also achieve low HPN-related complication rates and readmissions. Furthermore, collaboration between an IF center, specialist oncology center and local homecare nursing staff or relatives facilitating HPN administration can result in care provision over a large geographical area without compromising patient outcomes. This is supported by the fact that HPN complications, readmissions and overall survival were not affected by the distance from the patient’s home to the IF center. Efforts to streamline and improve coordination between the supra-regional and local centers could, therefore, enable HPN provision to more patients and help with the increasing demand for this treatment observed worldwide [8,20].

The main limitation of this study is that medical records were used as a data collection source. However, since the patient database was maintained prospectively, data completeness was continuously pursued. Nevertheless, data on changes in patients’ nutritional status, such as changes in weight, or performance status were not uniformly reported and, therefore, were not included in the analysis. Moreover, due to the retrospective nature of this study, we were not able to measure patient-reported quality of life using established questionnaires, which should be the focus of further prospective research.

## 5. Conclusions

In conclusion, we demonstrate one of the largest single-center experiences describing the service provision of HPN to patients with advanced cancers. Our study shows that establishing centralized care could lead to the provision of HPN to a large number of patients over a substantial geographical area while maintaining low complication and readmission rates and good quality outcomes for affected patients.

## Figures and Tables

**Table 1:** Patient demographics and clinical characteristics.

Patient Characteristics	N = 126 ^1^
**Age**	58 (49–69)
Sex	
Female	94 (75%)
Male	32 (25%)
**Indication for HPN**	
Bowel obstruction	117 (93%)
High output stoma	6 (4.8%)
Malnutrition	1 (0.8%)
Dysmotility	1 (0.8%)
Short bowel syndrome	1 (0.8%)
**Malignancy**	
Ovarian	54 (43%)
Pseudomyxoma peritonei	18 (14%)
Colorectal	14 (11%)
Gastric	10 (7.9%)
Small bowel adenocarcinoma	6 (4.8%)
Appendiceal	4 (3.2%)
Lymphoma	4 (3.2%)
Bladder	3 (2.4%)
Oesophageal	3 (2.4%)
Breast	2 (1.6%)
Endometrial	2 (1.6%)
Pancreatic	2 (1.6%)
Unknown primary	2 (1.6%)
Fallopian tube	1 (0.8%)
Lung	1 (0.8%)
**Body mass index (kg/m^2^)**	22.9 (20.3, 26.1)
**Venting gastrostomy present**	60 (48%)
**Number of HPN nights per week**	
5	2 (1.7%)
6	1 (0.9%)
7	114 (97%)
(Missing)	9
**Average daily kcal from HPN (kcal)**	1711 (255)
**Average daily volume from HPN (mL)**	2172 (504)

^1^ Median (IQR); *n* (%); mean (SD). HPN, home parenteral nutrition.

**Table 2:** Catheter characteristics.

HPN Catheter Characteristics	N = 126 ^1^
**CVC care**	
Homecare nurse	114 (90%)
Relative	10 (7.9%)
Self	2 (1.6%)
**CVC type**	
PICC	36 (29%)
Tunnelled	90 (71%)
**Lumen**	
Single	61 (48%)
Double	65 (52%)
**Concomitant SACT**	35 (28%)
**SACT via a distinct lumen of the HPN CVC**	13 (10%)

^1^ *n* (%); HPN, home parenteral nutrition; CVC, central venous catheter; PICC, peripherally inserted central catheter; SACT, systemic anti-cancer therapy.

**Table 3:** Univariate Poisson regression of the factors associated with CRBSI. SACT, systemic anti-cancer therapy.

Variable		Rate Ratio (95% CI, *p* Value)
Age at HPN initiation		0.956 (0.888 to 1.024, *p* = 0.204)
BMI at HPN initiation		1.127 (0.925 to 1.349, *p* = 0.203)
CVC lumen	Single	Reference
	Double	1.326 (0.176 to 12.796, *p* = 0.780)
SACT via HPN CVC	No	Reference
	Yes	2.775 (0.170 to 20.985, *p* = 0.359)
Venting gastrostomy present	No	Reference
	Yes	0.696 (0.072 to 5.273, *p* = 0.721)
Distance from patient home to IF centre	0–15 km	Reference
	16–30 km	0.264 (0.002 to 2.943, *p* = 0.378)
	>30 km	0.535 (0.041 to 3.873, *p* = 0.555)

**Table 4:** Univariate Poisson regression of the factors associated with mechanical CVC complications. SACT, systemic anti-cancer therapy.

Variable		Rate Ratio (95% CI, *p* Value)
Age at HPN initiation		1.005 (0.971 to 1.041, *p* = 0.795)
Sex	Female	Reference
	Male	0.611 (0.342 to 1.018, *p* = 0.074)
CVC type	PICC	Reference
	Tunnelled	0.489 (0.207 to 1.198, *p* = 0.107)
CVC lumen	Single	Reference
	Double	1.326 (0.176 to 12.796, *p* = 0.616)
SACT via HPN CVC	No	Reference
	Yes	0.408 (0.028 to 1.86, *p* = 0.361)
Venting gastrostomy present	No	Reference
	Yes	1.193 (0.496 to 2.94, *p* = 0.694)
Distance from patient home to IF centre	0–15 km	Reference
	16–30 km	1.658 (0.682 to 4.105, *p* = 0.263)
	>30 km	0.256 (0.039 to 0.984, *p* = 0.081)

## Data Availability

The data presented in this study are available on request from the corresponding author.
